# Supplementary material for: Factors Influencing Level and Persistence of Anti SARS-CoV-2 IgG after BNT162b2 Vaccine: Evidence from a Large Cohort of Healthcare Workers
Source: Vaccines (Basel). 2022 Mar 18;10(3):474. doi: 10.3390/vaccines10030474 (PMC8955419; doi:10.3390/vaccines10030474)

Table S1. Prevalence of seropositivity and median serological values among HCW of CSS participating to the serological post-vaccination survey on May 2021

	Number	Serological test positivity N (%)	IgG value (BAU/mL) (median, IQR)
**Participants**	**7729**	**7618 (98.6%)**	961 (523-1840)
Questionnaire filled	6824	6742 (98.8%)	968 (531-1850)
Questionnaire not filled	905	876 (96.8%)	918 (462-1780)
**Participants, with questionnaire filled, by vaccination against SARS-CoV-2 (up to May 2021)**			
Yes, 1 dose	132	131 (99.2%)	2080 (485-2080)
Yes, both doses	6562	6551 (99.8%)	982 (552-1840)
No	130	60 (46.2%)	17 (4.81-206)
**Participants included in analyses***	**6687**	**6675 (99.8%)**	990 (551-1870)

* with questionnaire filled, vaccinated by May 2021 and with a distance between vaccination and serological test >= 14 days

Table S2. Quintile distribution of serological values among the 6687 HCW of CSS participating to the serological post-vaccination survey on May 2021

Serological test values (quintile cut-off)	Number
<=475	1340
476-789	1336
790-1250	1351
1251-2079	1252
>= 2080	1408

Table S3. Multivariable ordinal logistic regression model (ORs and 95% CI) for predictors of lower serological values (quintiles of distribution) among vaccinated HCW of CSS.

	OR	95%CI	p-values
**Age (year)**	1.03	1.02-1.03	<0.001
**Gender**			
Female	1.00		
Male	1.17	1.05-1.31	0.005
**Job profile**			
Nurse	1.00		
Physician	0.99	0.87-1.12	0.876
Health care assistant (HCA)	0.97	0.83-1.14	0.738
Clinical staff (other than physician/nurse/HCA)	1.04	0.91-1.19	0.592
Administrative staff	1.00	0.85-1.19	0.959
IT/maintenance staff	0.99	0.75-1.29	0.919
**Smoking habit**			
Never smokers	1.00		
Former smokers	1.11	0.98-1.25	0.101
Current smokers	2.00	1.79-2.25	<0.001
**BMI**			
Normal weight (BMI18.5-25)	1.00		
Underweight (BMI <18.5)	1.13	0.91-1.42	0.264
Overweight (BMI 25-30)	0.90	0.81-1.01	0.074
Obesity (BMI 30-40)	0.89	0.75-1.04	0.144
Severe Obesity (BMI >40)	0.87	0.51-1.48	0.605
**AB0 blood group**			
0	1.00		
A	0.97	0.87-1.09	0.609
AB	1.01	0.82-1.25	0.926
B	0.93	0.80-1.09	0.393
Unknown	0.95	0.83-1.07	0.385
**Autoimmune diseases**			
No	1.00		
Yes	0.97	0.85-1.11	0.650
**Immuno-deficiency**			
No	1.00		
Yes	2.22	1.44-3.42	<0.001
**Hypertension**			
No	1.00		
Yes	1.00	0.87-1.14	0.998
**Diabetes**			
No	1.00		
Yes	1.02	0.75-1.38	0.924
**Cardiovascular diseases**			
No	1.00		
Yes	1.10	0.83-1.45	0.522
**Allergic rhinitis**			
No	1.00		
Yes	0.93	0.81-1.06	0.261
**Respiratory diseases**			
No	1.00		
Yes	1.14	0.85-1.53	0.378
**Kidney diseases**			
No	1.00		
Yes	1.30	0.72-2.34	0.377
**Neurological diseases**			

No	1.00		
Yes	0.75	0.41-1.36	0.344
**Neoplasms**			
No	1.00		
Yes	0.74	0.54-1.01	0.058
**Other chronic diseases**			
No	1.00		
Yes	1.16	0.81-1.67	0.410
**Previous SARS-CoV-2 infection***			
No	1.00		
Yes	0.13	0.11-0.15	<0.001
**Contacts with collegues in 2021**			
No	1.00		
Yes	1.15	1.04-1.27	0.007
**Contacts with inpatients in 2021**			
No	1.00		
Yes	1.01	0.92-1.12	0.790
**Household contacts in 2021**			
No	1.00		
Yes	0.73	0.62-0.87	<0.001
**Other contacts in 2021**			
No	1.00		
Yes	1.13	0.90-1.44	0.287
**Distance (week) between vaccination and serological test**	1.30	1.27-1.32	<0.001

**From March 2020 to the date of serological survey (May 2021).*

Figure S1. Flow chart of the study cohort of HCW of CSS

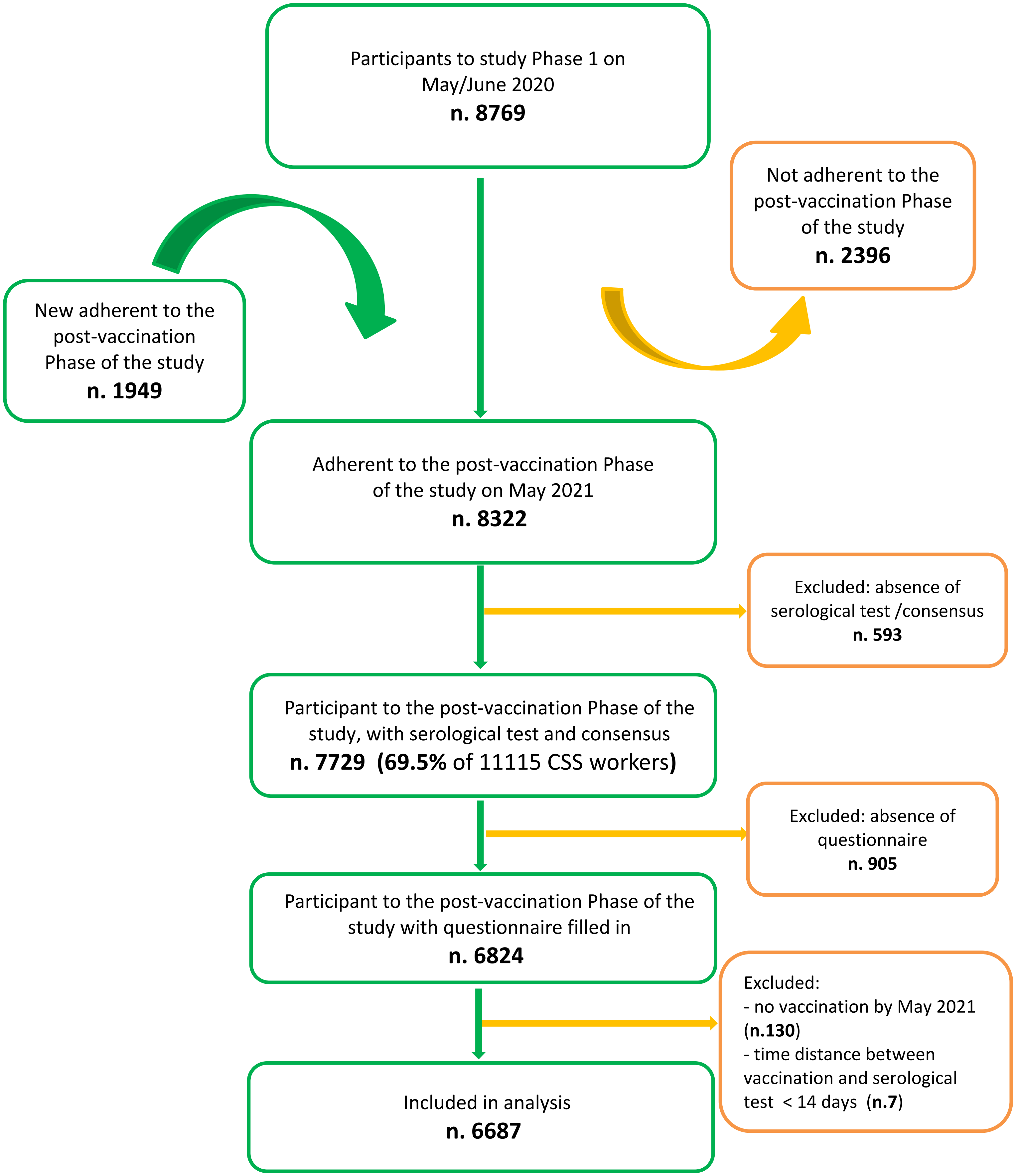

Figure S2. Box-plot of IgG values according to distance (months) between vaccination and serological test on May 2021, by self-reported immunodeficiency.

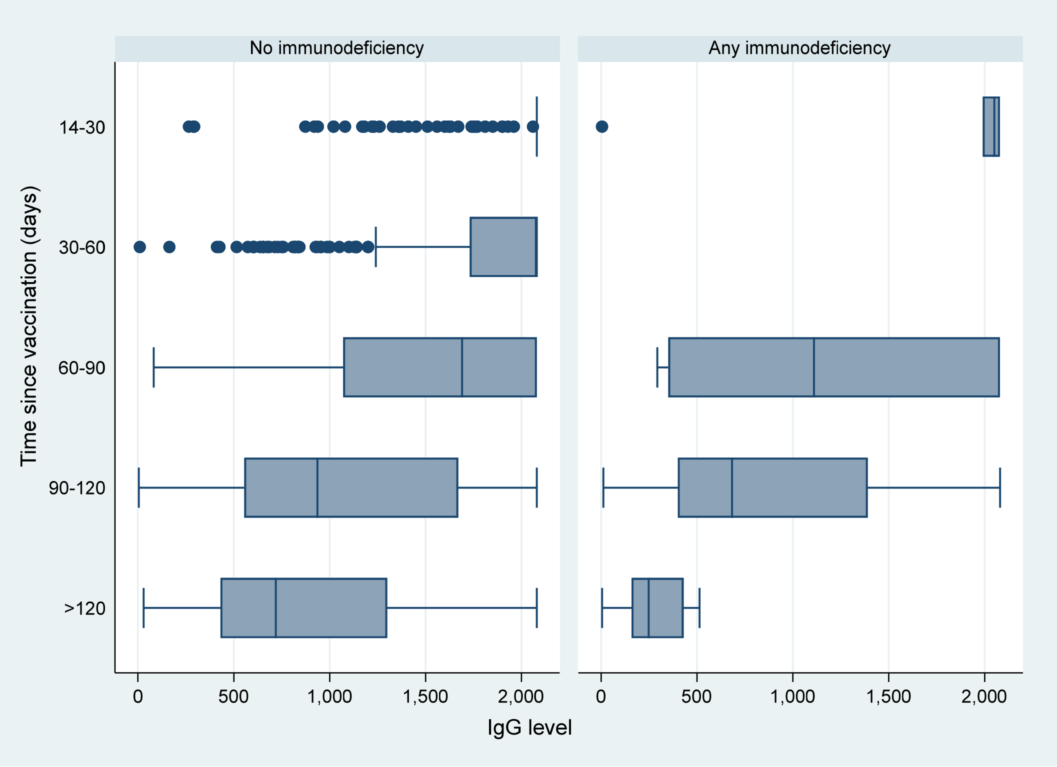

Supplement: Supplementary file 1 [file vaccines-10-00474-s001.zip › vaccines-1604245-supplementary.pdf]
